# Uncovering unique plasticity in life history of an endangered centenarian fish

**DOI:** 10.1038/s41598-020-69911-1

**Published:** 2020-07-30

**Authors:** Martin J. Hamel, Jonathan J. Spurgeon, Kirk D. Steffensen, Mark A. Pegg

**Affiliations:** 1 Warnell School of Forestry and Natural Resources, University of Georgia, 180 E. Green St., Athens, GA 30602 USA; 2 School of Agriculture, Fisheries, and Human Sciences, University of Arkansas at Pine Bluff, 1200 N. University Dr., Pine Bluff, AR 71601 USA; 3 Nebraska Game and Parks Commission, 3300 Holdrege St., Lincoln, NE 68503 USA; 4 School of Natural Resources, University of Nebraska-Lincoln, 3310 Holdrege St, Lincoln, NE 68503 USA

**Keywords:** Evolutionary ecology, Freshwater ecology, Population dynamics

## Abstract

The ability to adapt to changing environments is fundamental for species persistence. Both plasticity and genetic selection are potential drivers that allow for traits to be advantageous, thus leading to increases in survival or fitness. Identifying phenotypic plasticity in life history traits of long-lived organisms can be difficult owing to high survival, long generation times, and few studies at sufficient spatial and temporal scales to elicit a plastic response within a population. To begin to understand phenotypic plasticity of a long-lived freshwater fish in response to environmental conditions, we used a long-term data set consisting of over 1,200 mark-recapture events to inform our understanding of dynamic rate functions and life history attributes. Furthermore, we used a common garden experimental approach to confirm whether changes in life history traits are in response to plasticity in the reaction norm or are genetically derived. Using these approaches, we demonstrated differences in life history traits among Pallid Sturgeon (*Scaphirhynchus albus*) occupying river segments of varying physical and hydrological stress. The common garden experiment corroborated plastic phenotypic expression in reaction norms for age at first maturity, longevity, fecundity, and maximum size. These growth-mediated attributes resulted in differences in overall fitness traits, where Pallid Sturgeon fecundity was greater than a tenfold difference and 3–6 times the number of life-time spawning events. Anthropogenic modifications to river form and function are likely responsible for the variation in life history attributes resulting from an increased metabolic demand for maintaining station, foraging, and migration. Collectively, our approach provided surprising insight into the capabilities of a centenarian fish to dramatically respond to a changing environment.

## Introduction

Phenotypic plasticity is the ability of an organism to display various phenotypes in the event of environmental change. Plasticity in life history traits allow organisms the ability to adapt to stochastic conditions by matching growth, maturity, and longevity to in situ biotic or abiotic conditions, thereby providing the most advantageous strategy for completing its life cycle and contributing progeny^1,2^. Reaction norms are genetically based gradients of life history traits that can be expressed across environmental factors such as temperature or predation^3–5^. Estimating the reaction norms for phenotypic traits is important for understanding how populations function across spatial scales, calibrating size- and age-structured population models, and estimating species persistence to disturbance. Plasticity is adaptive when environments fluctuate temporally or spatially but can be maladaptive when the costs of plasticity outweigh its potential benefits or when selection narrows the norm of reaction^1,6,7^. For example, Trinidadian guppies displayed plastic responses in fitness metrics when exposed to predation, but those characteristics were selected for and became inherent in subsequent generations thereby shifting both life history traits and reducing future plasticity^5^. Thus, phenotypic plasticity and possible subsequent genetic selection may allow for increased survival and fitness in novel environments but may lead to reduced adaptability through time. Therefore, knowing the ability of species to adjust (phenotypic plasticity) or adapt (genetic selection) to large-scale anthropogenic changes in environments is fundamental for species persistence and maintaining biodiversity within the Anthropocene.

Changes in life history traits in response to harvest or environmental change have been documented for several species of fish [^8,9^, among others]. For example, various stocks of Atlantic Cod (*Gadus morhua*) have shown marked changes in age at sexual maturation, fecundity, and longevity in response to commercial over-harvest^9^. Moreover, anthropogenic alterations to aquatic environments have been linked to both community shifts in life history strategies [e.g.,^10^] and within-population variation in life history traits among varying environments^11^. Environmental disturbance can cause acute mortality, create additional stressors, or change resource allocation^1,12^, these effects can institute phenotypic changes in the population through increased mortality, physiological allostasis, or resource limitations^13,14^. Ultimately, growth rates are impacted by environmental disturbances and are the physiological mechanism for which variation in life history traits exist^15–17^. Faster growth early in life—whether density-dependent or density-independent—is how earlier sexual maturation can be attained and ultimately, a reduction in overall size and longevity^9,11^.

Common garden experiments help researchers understand the complex role among phenotype, genotype, and the environment. Typical common garden experiments occur where different populations are reared under the same environmental conditions to reduce the effect of environmental differences (e.g., latitude, altitude, or climate) on the phenotype, thus subsequent phenotypic changes that persist are assumed to be genetic^18,19^. Theoretically, the reciprocal experiment—where the same population of fish are exposed to different environmental conditions—can help to elucidate whether changes in life history traits are in response to plasticity in the reaction norm or are genetically derived. Challenges in these designs are that laboratory settings may not replicate cumulative environmental effects^20^ whereas *in natura* experiments may be difficult to assess due to low capture probabilities or the ability to control environmental factors^21^. Therefore, observing phenotypic plasticity and understanding factors responsible for shifts in life history traits in natural populations is challenging due to the inherent difficulties in experimental scope (large size of an experiment) and replication inasmuch as environmental constraints^22,23^.

Plasticity of reaction norms is unknown for long-lived fishes with specialized traits adapted from long-term survival in predictably variable environments (i.e., periodic strategists^24^. Periodic strategists pose challenges in determining the benefits of local adaptations or plasticity because generations of these species may span a century or more and are typically less abundant than short-lived generalist species^25,26^. With global environmental change—such as physical habitat modification and climate change—occurring at an expedited rate, understanding the capacity of how long-lived fishes may respond or adapt is imperative^27^. Population introductions of long-lived fish with known phenotype and genotype is key for the examination of reaction norms from individuals subject to selective pressures (environmental change). Results from common garden experiments such as this allow one to determine plasticity in traits and to verify key measures of population dynamics (e.g., growth rate). The goal in this study was to assess how genetic selection or phenotypic plasticity can explain differences in key life history traits (i.e., age and size at maturation, longevity, and reproductive fitness) to variation in altered riverine landscapes—the Missouri River. We quantified key dynamic rate functions (i.e., growth and survival) of Pallid Sturgeon (*Scaphirhynchus albus*)—a long-lived periodic strategist—throughout the Missouri River using mark-recapture data and a common garden experiment. The Missouri River has undergone varying levels (i.e., both spatially and temporally) of anthropogenic alteration over the last 70–90 years and it is unlikely that a single phenotype will result in the greatest fitness throughout the river system. Therefore, we predict that highly altered segments of the Missouri River will result in a reduction of various fitness traits compared to segments that mimic historical conditions.

## Background

### Pallid sturgeon life history

Pallid Sturgeon is a long-lived periodic-strategist that delays maturation and reproduction for several years—in some cases 10–20 years. Successive spawning events are infrequent and successful year classes likely occur only during years of optimal hydrological and climatological conditions. Pallid Sturgeon undergo ontogenetic shifts in diet where juveniles consume various macroinvertebrates^28,29^ and then begin to consume benthic fishes (primarily Cyprinids) following sexual maturation^29,30^. Early literature suggests that Pallid Sturgeon live to 41 y of age ^31^, but those estimates are based off age assignments from annuli deposition on pectoral fin rays which have been shown to provide inaccurate results^32,33^. Recent analyses utilizing bomb radiocarbon has indicated that Pallid Sturgeon from the upper Missouri River obtain an age of at least 65 years^34^. Pallid Sturgeon are estimated to become sexually mature from 10 to 17 years for females and 7 to 10 years for males and both sexes display an intermittent spawning cycle^35^.

### Distribution

**Figure 1:**
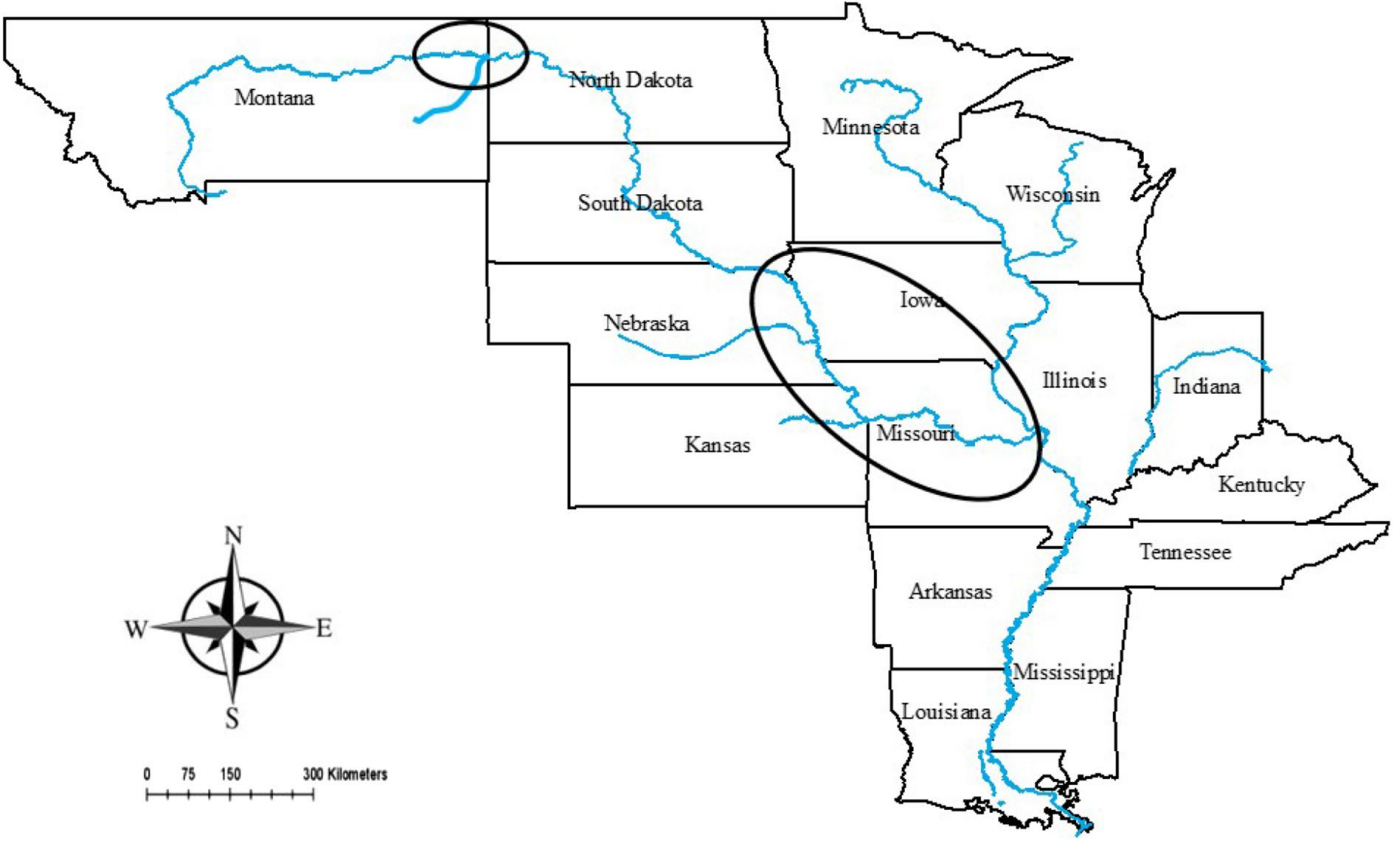
Map of the Missouri River basin differentiating between the upper basin (upper circle) and the lower basin (lower circle).

Pallid Sturgeon was classified as a U.S. federally endangered species in September, 1990 due to habitat loss from reservoir construction and channelization^36^. Pallid Sturgeon are currently distributed throughout the upper and lower basins of the Missouri River (Fig. 1). In the upper Missouri River, fish are distributed in the riverine portions between reservoirs and within the lower Yellowstone River^37,38^. Pallid Sturgeon found in this region (hereafter referred to as Upper Basin,UB) are either large and presumably old fish that were likely present prior to dam construction, or hatchery propagated individuals that were stocked beginning in the late 1990s. In the lower Missouri River (below the lowermost reservoir dam,Gavins Point dam, South Dakota, USA), Pallid Sturgeon are present throughout the entire 1,305 km of unobstructed Missouri River to its confluence with the Mississippi River. Pallid Sturgeon in this reach of river (hereafter referred to as lower basin; LB) are generally smaller, where the large, relic Pallid Sturgeon (such as those found in the UB) are not present. However, wild Pallid Sturgeon of unknown age and origin exist, as well as hatchery progeny that were similarly stocked beginning in the late 1990s.

### Environments

#### Upper basin

Hospitable habitat in the UB consists of inter-reservoir reaches of free flowing (i.e., lack of channel control structures) river and large tributary inputs (i.e., Yellowstone River, Montana, USA). The unaltered Missouri River between Fort Peck dam and the headwaters of Lake Sakakawea is a 327 km stretch of river. Water temperature, turbidity, and flow dynamics are altered from deep-water releases from Fort Peck dam^39,40^, but river form resembles historic conditions below the confluence with the Yellowstone River (rkm 2,525.4)^37^.

#### Lower basin

The lower Missouri River is primarily channelized containing a swift, deep channel that is operated by the U.S. Army Corps of Engineers for navigation. However, immediately below Gavins Point dam, the first 95 km of river is void of channel maintenance structures and resembles historical physical conditions (e.g., braided channel and exposed sand bars) despite a 77% reduction in the number and area of historic side channels^41^. Flow and water chemistry are altered in this stretch of river as a result of deep-water releases from Gavins Point dam^42^. The remaining Missouri River downstream to the mouth is channelized, where a 3.2 m thalweg is maintained and operated for tug-boat navigation.

### Hatchery propagation

A major component to the Pallid Sturgeon recovery program was the creation of a propagation and augmentation program^36^. Wild origin Pallid Sturgeon are captured and transported to hatchery facilities to spawn in controlled environments. Progeny are reared to increase survival and later individually tagged (i.e., PIT tags) and released at various stocking locations to supplement the natural population. Protocols are in place to maintain a genetically diverse program and to categorize each individual fish through unique markings or tags^43^. Tag returns thus provide important information on genetics, movement, population dynamics, and abundance.

Propagation for Pallid Sturgeon began in 1992; however, large-scale stocking didn’t occur until 1999. From 1999 to 2007, all Pallid Sturgeon stocked into the UB and LB were produced from adult fish that were collected from the UB. In 2008, the Pallid Sturgeon recovery team enforced a moratorium on stocking hatchery-reared Pallid Sturgeon of UB origin into the LB for fear of genetic mixing with those that might immigrate to the Mississippi River. Therefore, stocking protocols established prior to 2009 unintentionally set up a common garden experiment where hatchery progeny produced from the same UB fish genetics were stocked into the UB and LB.

## Methods

A multi-agency long-term monitoring program routinely sampled the riverine portions of the UB and LB of the Missouri River from 2006 to 2016. Collection efforts included sampling with standardized sampling gears and effort during the spring, summer, and fall. See Wildhaber et al.^44^ for a complete description of sampling protocols. A fin clip tissue sample was collected from all unmarked Pallid Sturgeon captures to genetically identify origin (e.g., hatchery-reared vs. wild) and year class through analysis of microsatellite loci or mitochondrial single nucleotide polymorphism assays^45,46^. Length (fork length), weight (g), and tag number were routinely recorded on collected specimens.

### Age and growth

Age and growth of Pallid Sturgeon were needed to characterize important life history traits, such as size at maturation, maximum size, and longevity. Growth of Pallid Sturgeon was assessed as the increase in somatic growth (i.e., fork length) between capture events for each individual within a basin. A total of 1,264 individual mark-recapture events from both wild and hatchery-origin fish that were at-large for at least 30 days were included in this analysis. Annual growth increments of tagged individuals were calculated from the following equation:

$${G}_{i}=\frac{\left({L}_{r}-{L}_{t}\right)}{Y}$$

where *G*_*i*_ is growth for fish *i*, *L*_*t*_ is fork length at tagging, *L*_*r*_ is fork length at recapture, and *Y* is the number of years between capture events. Annual growth increment per 50 mm length bin was plotted by the initial length at first tagging to determine the size at which growth begins to asymptote for Pallid Sturgeon located within each basin.

Hatchery propagation and subsequent stocking of uniquely tagged fish has provided an important data set of relatively young, known-age fish. However, age information is still lacking for wild-originated fish, particularly long-lived adults. To resolve this data gap, age for Pallid Sturgeon of any given size was estimated with von Bertalanffy growth curve parameters derived from mark-recapture data ^47^. We used a modification of the Fabens ^48^ method where growth increment data were fitted to the von Bertalanffy growth curve reformulated to account for observed growth between capture periods, so that

$$\Delta L=\left({L}_{\infty }-{L}_{t}\right)\left(1- {e}^{-kT}\right)$$

where Δ*L* is the increase in length between capture events, *t* is time of tagging, *T* is the number of years between tagging and recapture, ${L}_{\infty }$ is the von Bertalanffy length at infinity, and *k* is the von Bertalanffy growth rate coefficient. Parameters (± SE) for the von Bertalanffy growth curve were estimated iteratively using nonlinear regression (Gauss–Newton algorithm ^49^. An estimate of the time at length zero (*t*_*0*_) cannot be estimated with this method; therefore, we used the formula provided by Pauly ^50^:

$$\log \left( { - t_{0} } \right) = - 0.3922 - 0.2752\;log\;L_{\infty } - 1.038\;log\;k$$

Age (*t*) for Pallid Sturgeon of any given size (*L*_*t*_) could then be estimated by using a reformulation of the von Bertalanffy Eq. ^51^:

$$t={t}_{0}-{log}_{e}\left[(1-{L}_{t}/{L}_{\infty })/k\right]$$

A growth curve was then generated with the von Bertalanffy growth parameters, predicted ages, and fork lengths of Pallid Sturgeon for each basin so as to compare length-at-age differences and to infer life history characteristics (i.e., maximum age and size, length at sexual maturity). Information about the age distribution could then be incorporated into estimates for survival and life expectancy. Total annual survival (*S*) was estimated with Hoenig’s estimator^52^:

$$S = {1}-{4}.{22}/T_{max} ,$$

where *T*_*max*_ is the maximum age of fish within the population. Mean life expectancy was then calculated using the equation from Seber^53^

$$e_{0} = - 1/\ln \left( S \right),$$

where *e*_*0*_ is the mean life expectancy and *S* is annual survival. Data computations were performed using SAS software, version 9.4 of the SAS System (SAS Institute, Inc., Cary, North Carolina USA). Figures were created using SigmaPlot version 14, from Systat Software, Inc., San Jose California USA (www.systatsoftware.com).

### Common garden experiment

We examined hatchery-reared Pallid Sturgeon recaptures from the 2002 through 2008 year classes. These age groups represent fish that were produced from UB broodstock and stocked into both the UB and LB of the Missouri River. Additional year classes from earlier stockings (1997, 1998, 1999, and 2001) were included in the UB to increase sample size. There were 119 mark-recapture records of hatchery-reared fish from the UB and 355 hatchery-reared fish from the LB. Hatchery-reared Pallid Sturgeon were uniquely tagged prior to release and this information was utilized to determine hatch date, age, and stocking location. Fork length at age was compared among basins to examine differences in growth. Because these fish all come from UB broodstock, these results will be indicative of environmental effects on growth without confounding genetic influence.

### Reproductive information

The Pallid Sturgeon propagation and augmentation program annually collects adult size fish and assesses them for reproductive readiness. Pallid Sturgeon were assessed with ultrasound and endoscopology and those that were deemed reproductive were housed in hatchery facilities throughout the spawning process. Hatchery personnel recorded length, weight, and tag number (either new or old) prior to spawning. When spawning occurred, the number of eggs removed were recorded. Although this metric was not a measure of true fecundity, we assumed it was an accurate reflection of the reproductive potential of gravid female Pallid Sturgeon. Reproductive information was provided from Gavins Point National Fish Hatchery (Yankton, South Dakota, USA) in the LB and Garrison National Fish Hatchery (Bismarck, North Dakota, USA) in the UB.

### Ethics statement

Data collected for this study was from multiple U.S. state and federal agencies cooperatively working under the guidelines of the Pallid Sturgeon Population Assessment Program funded through the U.S. Army Corps of Engineers. All collection efforts followed the Pallid Sturgeon Handling Protocols approved by the U.S. Fish and Wildlife Service under the guidance of the Pallid Sturgeon Recovery Team. Each cooperating agency possessed independent federal and state endangered species collection permits required to obtain and possess Pallid Sturgeon.

## Results

### Common garden experiment

**Figure 2:**
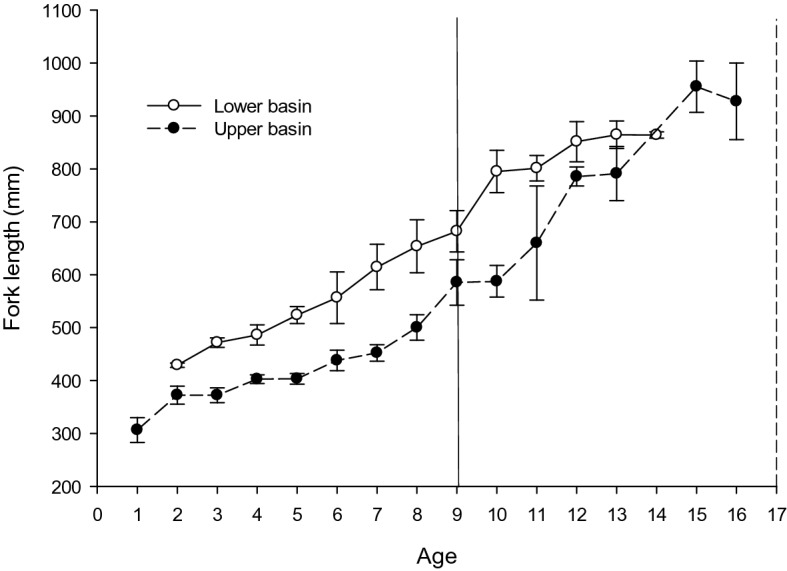
Mean length at age (± SE) for known-age hatchery-reared Pallid Sturgeon. All hatchery-reared fish were produced from adult broodstock collected in the upper basin and subsequently stocked into both the upper and lower basins of the Missouri River. Vertical lines represent minimum age at maturity reported from hatchery personnel in the lower basin (solid line) and upper basin (dashed line).

**Figure 3:**
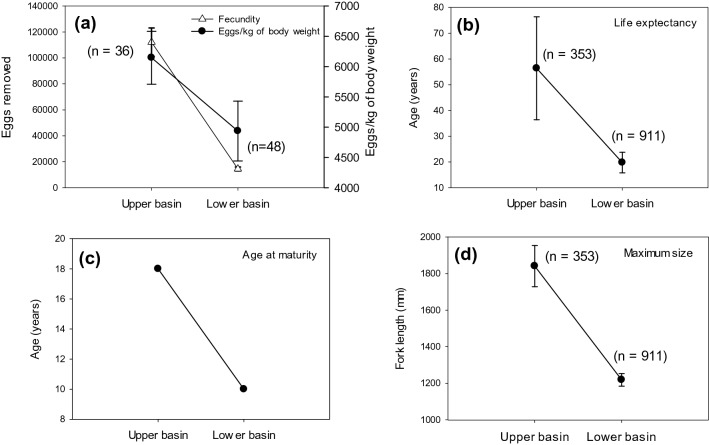
Reaction norms for selected life history traits from Pallid Sturgeon collected in the upper and lower basins of the Missouri River. Bars indicate standard errors. Fecundity (**a**) is reported as the number of eggs removed during egg extraction for hatchery propagation. Life expectancy (**b**) was calculated following methods outlined in Seber (1982). Age at maturity (**c**) was reported as the youngest known-age female in reproductive condition brought to a hatchery for reproductive assessments. Maximum size (**d**) was calculated from mark-recapture data (see methods). Panels (**a**) and (**c**) were data collected from wild-origin Pallid Sturgeon broodstock used for hatchery propagation. Panels (**b**) and (**d**) came from the mark-recapture data set including all hatchery and wild-origin Pallid Sturgeon.

Mean length at age for hatchery-reared Pallid Sturgeon was markedly different between the UB and LB, despite hatchery-reared progeny originating from the same UB parentage (i.e., stocked into both the UB and LB). Pallid Sturgeon in the LB were larger at a given age until age-14 (Fig. 2). The growth trajectory of Pallid Sturgeon from the LB was steep until age-10 (approximately 800 mm), which is the size and age when hatchery personnel begin seeing fish become reproductively viable (Steffensen et al. 2013). After age-10, somatic growth abruptly declines where some fish grow little throughout the remainder of their life. For example, an adult Pallid Sturgeon recaptured in the LB in 2013 that had been at-large for 12 years displayed an annual growth rate of 6–7 mm per year. In the UB, the observed length at age was comparatively smaller until age-14, but growth continued on an upward trajectory beyond the inflection point of reduced growth for stocked fish from the LB. Collectively, these data indicate plasticity in the reaction norm of dynamic rate functions in hatchery-reared fish linked to the same parental group originating in the UB (Fig. 3).

### Age and growth

**Figure 4:**
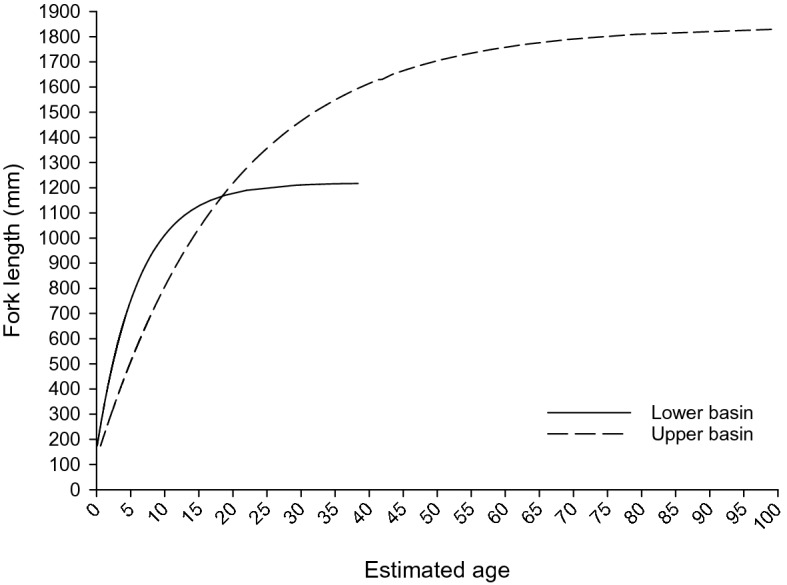
Estimated age at fork length (FL) for Pallid Sturgeon captured in the upper and lower basin of the Missouri River. Growth increment data were fitted to a von Bertalanffy growth curve reformulated in terms of the increment of growth and the period of time between captures. Age for fish of any given size (FL) was estimated with parameters derived from tagging data.

Analyses combining hatchery-reared and wild origin Pallid Sturgeon (n = 1,264) provided a means to characterize life history traits. Pallid Sturgeon displayed marked differences in maximum size, lifetime growth trajectories, and reproductive characteristics between the UB and the LB. Upper basin Pallid Sturgeon grew slowly throughout life, but that growth persisted until fish obtained large sizes and old ages. Conversely, Pallid Sturgeon from the LB grew relatively quickly at early ages (i.e., juvenile stage) until sexual maturation presumably occurred. Pallid Sturgeon thereafter grew little, corresponding to the reduced size in maximum length and overall longevity (Fig. 4). Assuming environmental influences heavily outweigh genetic disposition in wild-origin fish similar to what was discovered in our common garden experiment, we witnessed significant plasticity in reaction norms of life history traits (Fig. 3). Maximum size estimated from L-infinity was 1,219 mm (34.56 SE) and was corroborated from field observations of the largest fish ever collected during field sampling (1,197 mm) (Fig. 3d). Estimated age from mark-recapture data suggests that maximum expected age in the LB population was 39 years (95% CI 34–49). Pallid Sturgeon growth in the UB was characterized by a greater overall maximum length and increased longevity. The L-infinity parameter indicated a maximum size of 1,841 mm (112.80 SE) (Fig. 3d) and estimated age from mark-recapture data suggests that maximum expected age in the UB population was 100 y (95% CI 83–120).

Hatchery personnel reported female Pallid Sturgeon in the LB becoming sexually mature as early as age-10^46^ (Fig. 3c,Table 1) and at an approximate length of 750 mm. Female Pallid Sturgeon in the UB became sexually mature at age-18^47^ (Fig. 3c) and at an approximate length of 950 mm. The mean number of eggs extracted for spawning from female Pallid Sturgeon varied dramatically among basins and was related to the overall size (i.e., weight) of fish, where female Pallid Sturgeon from the UB weighed on average 21.6 (± 0.65) kg and female Pallid Sturgeon from the LB weighed on average 3.1 (± 0.17) kg (Table 1). Hatchery personnel extracted a mean of 121,175 (± 10,932) eggs (Fig. 3a) from female Pallid Sturgeon from the UB. In the LB, a mean of 14,531 (± 1,484) eggs (Fig. 3a) were extracted (Table 1). Recaptures of individual fish and assessing reproductive condition in subsequent years indicate that female Pallid Sturgeon spawn on a cycle of 2–3 years in the UB and 1–3 years in the LB^54,55^.

**Table 1:** Differential reproductive and life history traits among Pallid Sturgeon from the upper and lower basins of the Missouri River, USA.

Trait	Upper Basin	Lower Basin
Female weight (kg)	21.6 ± 0.7 kg	3.1 ± 0.2 kg
Eggs/kg of body weight	6,146 ± 437	4,936 ± 404
Eggs extracted	112,175 ± 10,932	14,531 ± 1,484
Age at first maturity	17 female; 10–17 male	10 female; 7–10 male
Spawning cycle	2–3 years	1–3 years
Maximum age	100 years (95% CI 83–120 years)	39 years (95% CI 34–49 years)
L^∞^	1,841 mm (95% CI 1618–2062 mm)	1,219 mm (95% CI,1,151–1,287 mm)
Annual survival	96% (95% CI 95–97%)	89% (95% CI 87–91%)
Mean life expectancy	56.4 years (95% CI 45–76 years)	19.8 years (95% CI 17–24 years)

Reproductive information (mean ± SE) was collected from hatchery personnel during annual reproductive assessments for hatchery propagation; whereas, life history traits were calculated with parameters derived from mark-recapture data (95% CI).

Annual survival estimates ranged from 89% (95% CI 87–91%) in the LB to 96% (95% CI 95–97%) in the UB (Table 1). The mean life expectancy of Pallid Sturgeon in the UB and LB was 56 years (95% CI 45–76 years) and 20 years (95% CI 17–24) (Fig. 3b). Combining life expectancy with reproductive information resulted in significant differences in fitness tradeoffs. Upper basin Pallid Sturgeon would result in approximately 13–20 spawning cycles (at 2–3 years intervals) for adult female Pallid Sturgeon and 3–11 spawning cycles (at 1–3 years intervals) in the LB. Lifetime reproductive output for females from the UB would therefore equate to 1.5e^5^–2.4e^5^ eggs produced compared to 53,280–87,186 eggs in the LB.

## Discussion

Contrasting habitat conditions among two sections of a large-river system altered growth patterns, and subsequent life history traits, of a long-lived species. Phenotypic plasticity was evident in reaction norms for age at first maturity, longevity, fecundity, and maximum size (Fig. 3). These growth-mediated attributes resulted in differences in overall fitness traits, where differences in Pallid Sturgeon fecundity was greater than a tenfold difference and many more life-time spawning events. Although long-lived species pose several limitations on examining a full complement of life history attributes (e.g., longevity and maximum size), our common garden experiment provided a means to corroborate growth patterns – assessed with mark-recapture data—of natural fish that have only ever been present within their respective basins. Collectively, our approach provided surprising insight into the capabilities of a possible centenarian fish to dramatically respond to a changing environment.

Anthropogenic modifications to river form and function are likely responsible for the variation in life history attributes of Pallid Sturgeon. The LB of the Missouri River is largely a homogenized system of uniform depths and velocities. Channelization of the Missouri River begins at rkm 1,213 and consists of river control structures (i.e., various dike structures) on the inside bends and revetted rock that armors the outside bend. This river modification scheme was designed to create a self-scouring channel that supports tug-boat navigation (i.e., creates at least a 2.7-m deep channel). As a result, mean depth and velocity has greatly increased over historic conditions^39^. The lack of shallow, slow water velocity habitat has been identified as a major factor in the decline in Pallid Sturgeon abundance as it provides refugia from high velocity—for all ages—and allows drifting larvae to settle and grow during highly vulnerable periods of their life^36,37,56^. In the present day, shallow, slow water habitat that is necessary is maximized during the following rare events: (1) extremely low flow periods in the main channel and (2) extreme high flow periods that result in out-of-bank flow events that enter the floodplain^57^. We hypothesize that a lack of consistent in-channel velocity refugia is a major contributor in driving growth characteristics, ultimately responsible for earlier onset of sexual maturation and overall reduction in size and longevity. The increased metabolic demand for maintaining station, foraging, and migration create situations where allostatic overload may occur, particularly when faced with other physiological stressors such as temperature extremes or high pollutant loads^58^.

Slight genetic differences may occur among river basins but were not solely responsible for the differences in life history traits seen in this study. Few generations of fish—particularly in the UB—have occurred since the wide-spread transformation of the Missouri River, which explains the breadth in plasticity of reaction norms. However, it is likely that selective forces may lead to genetic differences in life history traits in the future as many studies have revealed rapid evolution in reaction norms^5,59–61^. Plasticity in reaction norms has likely been adaptive in the LB as multiple generations (i.e., recruitment) have occurred since transformation of the lower Missouri River in the 1950s. Plasticity in reaction norms can be maladapative though, particularly when there is increasing selection and eventual genetic change for that plastic trait. Earlier onset of maturation is a reproductive strategy initiated by early-life growth patterns that allows organisms to produce progeny as quickly in life as possible^15,62,63^. In the case of Pallid Sturgeon from the LB, earlier onset of maturation allows female Pallid Sturgeon to produce as many possible spawning opportunities in a shortened lifespan—especially in relation to Pallid Sturgeon from the UB. While this strategy can be advantageous if environmental conditions are suitable for reproduction and recruitment, there are several factors that are concerning for species persistence. Long-lived, periodic strategists often spawn intermittently or produce inconsistent recruitment patterns. This strategy is effective because of longevity—where successive failed recruitment during years of unfavorable environmental conditions are mitigated from the successful recruitment of only one or two strong year classes during favorable conditions. However, when longevity is substantially reduced, the number of opportunities for providing a successful recruitment year decrease. Furthermore, large-scale impacts to already perturbed systems may take multiple years to return to pre-existing conditions or reach a new ecological equilibrium. If anthropogenic change intensifies (e.g., climate change), plasticity and genetic adaptation may be pushed to their limits.

Phenotypic plasticity in reaction norms has not been documented in long-lived periodic strategists. This is perhaps owing to the difficulty in separating genetic from environmental influences on phenotypic variation throughout a long life-span with few observed generations. Our common garden experiment allowed us to confirm phenotypic variation in Pallid Sturgeon, where plasticity occurred in response to environmental change, shifting the phenotype along a norm of reaction^3,4,6^. Although there was substantial variation in plasticity of age at maturity, longevity, and maximum size, it is unknown as to how plastic the reaction norm is among individuals or populations. Recent work by Steffensen et al.^64^ has shown a continual decline in maximum size of recent year classes of hatchery-produced Pallid Sturgeon from the LB, indicating a much wider range in plasticity than anticipated. Future work elucidating the complete reaction norm slope or shape and how they differ among Pallid Sturgeon genotypes may aid in the recovery process and better inform population models attempting to determine population viability and likely responses to alternative management actions.
